# Improvement of atropine on esophagogastric junction observation during sedative esophagogastroduodenoscopy

**DOI:** 10.1371/journal.pone.0179490

**Published:** 2017-06-27

**Authors:** Zhihao Chen, Lingang Liu, Jiangfeng Tu, Guangming Qin, Weiwei Su, Xiaoge Geng, Xiaojun Chen, Hongguang Wu, Wensheng Pan

**Affiliations:** 1 Department of Gastroenterology, the Second Affiliated Hospital, School of Medicine, Zhejiang University, Hangzhou, China; 2 Department of Gastroenterology, Longsai Hospital, Ningbo, China; 3 Department of Gastroenterology, Zhejiang Provincial People’s Hospital, Hangzhou, China; 4 Department of Laboratory, the Second Affiliated Hospital, School of Medicine, Zhejiang University, Hangzhou, China; 5 Department of Gastroenterology, Quzhou Second People's Hospital, Quzhou, China; University Hospital Llandough, UNITED KINGDOM

## Abstract

**Background and study aims:**

Although sedation esophagogastroduodenoscopy (EGD) is now widely used, previous research has reported that sedation during EGD exhibits a negative effect on esophagogastric junction (EGJ) exposure. Atropine might improve EGJ exposure, as noted in clinical practice. The aim of this study was to examine whether sedation had a negative effect on EGJ observation in the Chinese population, and whether atropine had some ability to act as an antidote to this unexpected secondary effect of sedation.

**Patients and methods:**

In this cross-sectional study, subjects were divided into the following three groups according to the methods of EGD examination: the non-sedation group, the propofol-fentanyl combined sedation group and the combined sedation with atropine administration group. The EGJ observation was assessed by a key photograph taken with the endoscopic camera 1 cm from the EGJ, which was rated on the following four-degree scale: excellent (score = 4), good (score = 3), fair (score = 2) and poor (score = 1).

**Results:**

The EGJ exposure was better in the sedation group administered atropine (score = 2.64±1.05) than in the sedation group (score = 1.99±1.08, P<0.05) but not as good as in the non-sedation group (score = 3.24±1.12, P<0.05). Reduced detection of EGJ diseases in the sedation group was also found, compared to the non-sedation group (P<0.05). Only the use of atropine (OR = 2.381, 95%CI: 1.297–4.371, P = 0.005) was independently associated with excellent observation of the EGJ during sedation EGD.

**Conclusions:**

Combined propofol-fentanyl sedation reduces the extent of exposure of the EGJ during EGD and reduces the detection of EGJ diseases. The application of atropine in the sedation endoscopy examination helped to achieve better EGJ observation, but still cannot achieve an equal extent of exposure compared to non-sedation EGD.

## Introduction

Esophagogastric junction (EGJ) diseases require careful clinical attention because of their relatively high prevalence. Although the global incidence of gastric cancer has decreased due to the reduction in distal cancers[1], adenocarcinoma of the EGJ has shown a rapidly increasing trend in recent years, in both the West[2] and the East[3, 4]. This increase may continue in the future.

Currently, esophagogastroduodenoscopy (EGD) is an efficient, sensitive and economical form of examination for the detection of upper gastrointestinal diseases. It has also been accepted as the most helpful diagnosis and surveillance tool for adenocarcinoma of the EGJ or esophagus and its possible precursor, Barrett’s esophagus[5–8].

With the maturation of sedation technology, painless endoscopy has been proven to result in higher physician satisfaction and better outcome of the endoscopic procedure[9]. Thus, the majority of EGD procedures are performed with sedation in United States and some European countries[10, 11]. Endoscopic sedation is also in increasingly common use in China.

However, there remain some defects of painless esophagogastroduodenoscopy. It has been noted that sedation is related to a variety of adverse cardiopulmonary events such as hypoxia, hypotension and arrhythmia[12]. However, the negative impact of sedation on the EGD detective efficacy must also be taken seriously, particularly the unexpected effect reported on EGJ observation [13].

Muscarinic acetylcholine receptor antagonists are regularly used during endoscopy such as Scopolamine Butylbromide which is standard, independently of weight or other patients’ conditions, to inhibit gastrointestinal peristalsis and reduce mucus secretion, thereby providing a better view[14]. Atropine is most commonly used in China. Furthermore, in recent years of clinical practice, we have observed another delightful effect of atropine: improved EGJ observation seemed to accompany the application of atropine.

Therefore, we wanted to examine whether sedation had a negative effect on EGJ observation in the Chinese population and whether atropine had some antidote ability against this unexpected secondary effect of sedation.

## Patients and methods

### Selection of patients

In this cross-sectional study, the subject population consisted of both inpatients and outpatients admitted to the Second Affiliated Hospital of Zhejiang University School of Medicine for esophagogastroduodenoscopy from April 2014 to June 2016. The inclusion criteria were as follows: scheduled for diagnostic upper gastrointestinal endoscopy for the evaluation of dyspeptic complaints, aged over 18 years, and underwent either non-sedation EGD or sedation EGD with or without atropine. 411 participants met with inclusions criteria. The exclusion criteria were as follows: past history of malignancy, acute critical illness or organ dysfunction, heavy drinking, pregnancy and age over 85 years. 15 subjects were excluded who met with at least one item of the exclusion criteria. The subjects’ basic information including sex, age, height, weight and comorbidity was recorded, together with medical record numbers that could identify individual subjects. 6 Subjects without complete baseline demographic or clinical information were also excluded before statistical analysis. The final total sample size was 390. Original information of subjects can be inferred to the S1 Table.

Subjects underwent different methods of EGD examination based on their individual patient willing, and accordingly divided into three groups: the non-sedation group(n = 99), the propofol-fentanyl combined sedation group(n = 203) and the combined sedation with atropine administration group(n = 88). According to the sample size estimation based on preliminary data from our early observation of 30 subjects (10 of each group), with a two-tailed test of α = 0.05, 1 – β (the power) = 0.90, and 5% drop-out rate, at least 53 patients in each group were required. Thus the sample size of this study met with the requirement.

Subject information was recorded in three groups according to the methods of EGD examination: the non-sedation group, the propofol-fentanyl combined sedation group and the combined sedation with atropine administration group. All subjects provided written consent, and the study was reviewed and approved by the institutional review board (IRB) of the Second Affiliated Hospital of the Zhejiang University School of Medicine (2016–023).

### Endoscopy and sedation procedure

All EGD examinations in this study were performed by the same experienced gastroenterological endoscopist (WS Pan), using single-channel upper gastrointestinal endoscopes (GIF-H260, Olympus Company). All EGD examination procedures were executed in a standard manner according to the Guidelines for Gastrointestinal Endoscopy[14].

As a standard local laryngeal anesthesia method, patients of the non-sedation group received 10 mL of lidocaine jelly (Harvest Pharmaceutical Co. Ltd, Shanghai, China) before the procedure. The combined sedation with propofol and fentanyl in the two sedation groups was conducted by certified anesthesiologists under the supervision of the endoscopist according to the official protocol in China[15]: an initial dose of 50 μg fentanyl and 1.5 mg/kg propofol was administered until the patient reached a state of deep sedation, as defined by the Ramsay Sedation Scale[16]; in case of insufficient sedation, 0.2–0.5 mg/kg boluses of propofol were added. For the atropine plus combined sedation group, an intravenous bolus of 0.5 mg atropine (Tianjin Kingyork Group Co. Ltd, Tianjin, China) was administered 30 seconds after conscious sedation was achieved and before the EGD examination began.

**Fig 1:**
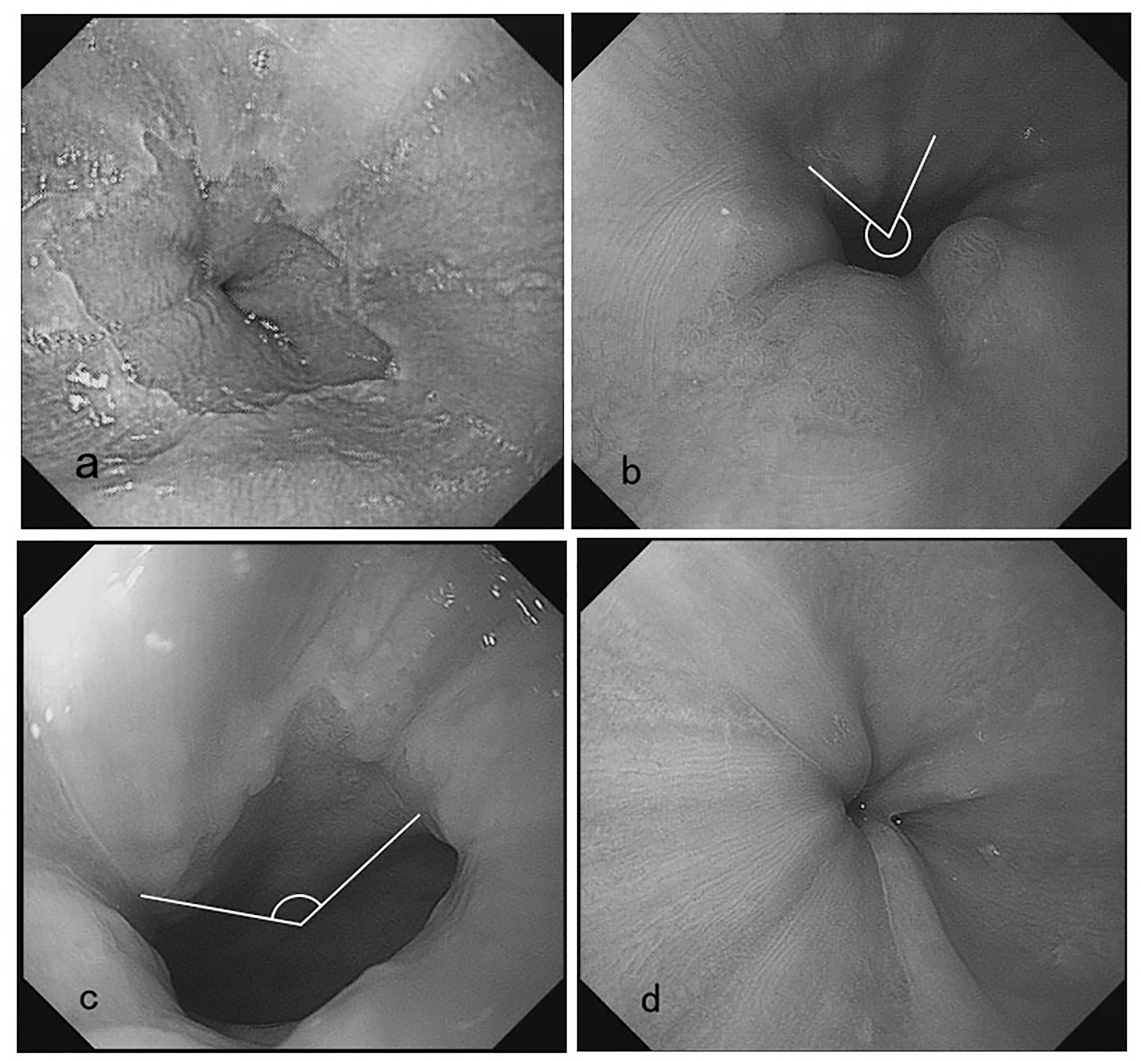
The esophagogastric junction exposure extent scale **(a).** Excellent (100% of the EGJ, score = 4) **(b).** Good (100% > EGJ ≥ 50%, score = 3) **(c).** Fair (50% > EGJ, score = 2) **(d).** Poor (EGJ not visualized, score = 1). White lines indicate the observation region.

The EGJ was defined as the proximal margin of the gastric mucosal folds[17]. The EGJ observation was assessed by the key photographs taken using the endoscopic camera 1 cm from the site of the EGJ during both insertion and withdrawal. The key photographs were not taken until the operator had inflated the esophagus and inspected the EGJ territory as clearly as possible. For statistical analysis, the extent of EGJ exposure was scored on a four-degree scale[13]: excellent (100% of the EGJ, score of 4), good (100% > EGJ ≥ 50%, score 3), fair (50% > EGJ, score 2), and poor (EGJ not visualized, score 1) (Fig 1). The EGJ exposure grading was all worked out by another experienced endoscopist(Wu HG) blindly who was not involved in gathering the images and was not aware of the patient’s method of EGD examination at the time of image capture.

During the sedation endoscopic procedure, all patients were monitored for oxygen saturation, pulse rate and arterial blood pressure by a monitoring instrument (Type: Ipm 12, Shenzhen Mindary Biomedical Electronic Co. Ltd, Guangdong, China).

### Statistical analysis

The statistical analyses were performed using the SPSS 18 (SPSS Inc. Chicago, USA) software. Continued variables were expressed as the means ± SD and were compared using one-way analysis of variance (ANOVA). The categorical variables were analyzed using Fisher’s exact test or the χ^2^ test. Logistic regression analysis was applied to find the independent factors associated with excellent observation, which showed a significant association in the univariate analysis. A value of *P* less than 0.05 was considered statistically significant.

## Results

A total of 390 patients were included in the study (99 non-sedation, 203 propofol-fentanyl combined sedation, 88 combined sedation with atropine administration). All the baseline demographic and clinical characteristics were recorded in three groups, and no significant difference was found among the three groups by the difference analysis (Table 1). No severe sedation-related cardiopulmonary adverse events such as hypoxia, hypotension and arrhythmias or explicit sedation-related symptoms like vomiting occurred during any endoscopy process, due to the professional sedation executed by experienced anesthetists and cautious monitoring.

**Table 1:** Baseline characteristics of subjects.

		Non-sedation(n = 99)	Sedation (n = 203)	Sedation with atropine(n = 88)	*P* value
Demographic characteristics					
	Age	50.09±14.87	49.24±12.54	48.83±13.76	0.800
	Male, n(%)	48(48.5%)	100(49.3%)	49(55.7%)	0.540
	Weight	62.53±10.96	62.56±10.17	62.39±10.26	0.992
	Height	165.49±7.13	164.93±7.66	165.90±7.36	0.562
	BMI	22.71±2.90	22.91±2.72	22.61±2.84	0.688
Comorbidity					
	Hypertension, n(%)	15(15.2%)	26(12.8%)	8(9.1%)	0.454
	Diabetes melitus, n(%)	7(7.1%)	6(3.0%)	5(5.7%)	0.240
	Hyperlipemia, n(%)	7(7.1%)	14(6.9%)	5(5.7%)	0.914
	Cardiovascular disease, n(%)	12(12.1%)	14(6.9%)	3(3.4%)	0.070
	Liver disease, n(%)	3(3.0%)	14(6.9%)	6(6.8%)	0.374
	Thyroid disorder, n(%)	5(5.1%)	17(8.4%)	4(4.5%)	0.367
	Psychiatric disorder, n(%)	6(6.1%)	8(3.9%)	2(2.3%)	0.422

All endoscopic disease detection was similar among three groups except that a significant reduction in carditis and hiatus hernia detection was observed during sedation EGD compared to non-sedation EGD (Table 2).

**Table 2:** Endoscopic findings during EGD.

Endoscopic findings	Non-sedation(n = 99)	Sedation (n = 203)	Sedation with atropine(n = 88)	*P* value
Carditis, n(%)*	35(35.4%)	33(16.3%)	10(11.4%)	<0.05
Hiatus hernia, n(%)*	11(11.1%)	4(2.0%)	4(4.5%)	<0.05
Esophagitis, n(%)	6(6.1%)	17(8.4%)	6(6.8%)	0.748
Gastric polyp, n(%)	8(8.1%)	34(16.7%)	10(11.4%)	0.095
Gastric ulcer, n(%)	3(3.0%)	8(3.9%)	1(1.1%)	0.445
Duodenal polyp, n(%)	0(0.0%)	3(1.5%)	1(1.1%)	0.485
Duodenal ulcer, n(%)	13(13.1%)	18(8.9%)	11(12.5%)	0.446
Neoplasm, n(%)^#^	1(1.0%)	3(1.5%)	5(5.7%)	0.055

EGD, esophagogastroduodenoscopy.

* *P*<0.05 for non-sedation vs. sedation group

# 9 patients with neoplasm: 8 gastric adenocarcinoma, 1 gastric mucosal-associated lymphoid tissue lymphoma.

The extent of exposure of the EGJ was significantly higher in the non-sedation group than in the sedation group, while the sedation group administered atropine had a significantly better view of the EGJ than the sedation group without atropine administration (non-sedation group score = 3.24±1.12, sedation without atropine 1.99±1.08, sedation with atropine 2.64±1.05, *P*<0.001, Table 3).

**Table 3:** Observed esophagogastric junction region exposure grade and score.

Exposure grade(Score)	Non- sedation(n = 99)	Sedation (n = 203)	Sedation with atropine(n = 88)	*P* value
Excellent(4), n(%)	64(64.6%)	39(14.3%)	25(28.4%)	
Good(3), n(%)	7(7.1%)	30(14.8%)	19(21.6)	
Fair(2), n(%)	16(16.2%)	53(26.1%)	31(35.2%)	
Poor(1), n(%)	12(12.1%)	91(44.8%)	13(14.8%)	
Score, Mean±SD*	3.24±1.12	1.99±1.08	2.64±1.05	<0.001

* *P*<0.001 for every two groups: non-sedation vs. sedation, sedation with atropine vs. sedation and non-sedation vs. sedation with atropine

Based on this result, we further explored its potential correlated factors by dividing all the subjects of sedation EGJ into two groups: excellent EGJ exposure (n = 54) and non-excellent EGJ exposure (n = 237). In the univariate analysis (Table 4), whether the EGJ exposure was excellent was related to two factors: atropine administration and the comorbidity of hypertension. A larger proportion of subjects with atropine administration achieved excellent-grade EGJ exposure compared to subjects without atropine (OR = 2.381, 95%CI: 1.297–4.371, *P* = 0.004). However, subjects with comorbid hypertension achieved excellent-grade EGJ exposure less frequently (OR = 0.246, 95%CI: 0.057–1.062, *P* = 0.043).

**Table 4:** Univariate analysis of factors associated with excellent exposure of esophagogastric junction territory during sedation EGD.

Variables		excellent(n = 54)	non-excellent(n = 237)	*P* value
Demographic characteristics				
	Age	51.93±12.24	48.48±12.98	0.076
	Male	25(46.3%)	124(52.3%)	0.424
	Weight	63.15±8.62	62.36±10.51	0.611
	Height	164.93±8.53	165.29±7.20	0.746
	BMI	23.21±2.66	22.73±2.86	0.253
Comorbidity				
	Hypertension, n(%)*	2(3.7%)	32(13.5%)	0.043
	Diabetes melitus, n(%)	3(5.6%)	8(3.4%)	0.448
	Hyperlipemia, n(%)	4(7.4%)	15(6.3%)	0.772
	Cardiovascular disease, n(%)	4(7.4%)	13(5.5%)	0.587
	Liver disease, n(%)	5(9.3%)	15(6.3%)	0.442
	Thyroid disorder, n(%)	3(5.6%)	18(7.6%)	0.601
	Psychiatric disorder, n(%)	3(5.6%)	7(3.0%)	0.344
Atropine, n(%)^#^		25(46.3%)	63(26.6%)	0.004

EGD, esophagogastroduodenoscopy.

* Odds Ratio for excellent exposure of (hypertensive/non-hypertensive)

= 0.246,95%CI: 0.057–1.062, *P* = 0.043.

# Odds Ratio for excellent exposure of (atropine administered/non-atropine)

= 2.381,95%CI: 1.297–4.371, *P* = 0.004.

To examine their independent effects on EGJ exposure, we performed multivariate logistic analysis of excellent or non-excellent EGJ exposure and the possible indicators. The administration of atropine (OR = 2.381, 95%CI: 1.297–4.371, *P* = 0.005) proved to be the only independent factor that contributed to excellent observation of the EGJ during sedation EGD.

## Discussion

The high incidence of EGJ diseases is indisputable, including many EGJ, gastric and esophageal diseases. As a specialized medical examination method, it is very important for endoscopy to be executed in the EGJ area with the best possible exposure. A previous study in Korea found that sedation with propofol during EGD had a significantly negative effect on EGJ/Z−line exposure compared to the non-sedation group[13], a phenomenon also observed in our clinic practice. We wanted to examine this relatively poor extent of exposure of EGJ during sedation EGD in the Chinese population. We also found a significant decrease in EGJ disease detection during sedation EGD, which may result from the poor EGJ exposure.

The extent of EGJ exposure is determined by several anatomical factors in the resting state. The dominant two mechanisms[18] are the pinchcock function of the diaphragmatic crura on the lower esophagus and the tone of the lower esophageal sphincter (LES). It is now generally believed that the diaphragm not only plays the role of a respiratory muscle with involuntary movement but also acts as a voluntary muscle. Therefore, during non-sedated EGD examination, endoscopic operators can pursue better exposure of the EGJ by asking the subjects to inhale deeply[19]. The LES, in contrast, is a group of involuntary smooth muscles. However, its resting tone is still regulated by a wide variety of neural, hormonal and drug influences. Especially for the sympathetic and parasympathetic nervous system, the LES is regulated by filaments from the vagus nerves[20, 21] and sympathetic filaments from the T6-T10 sympathetic ganglion[22, 23]. Other factors, including certain hormones such as gastrin, cholecystokinin, secretin,[24, 25] and glucagons or drug-like cholinergic and adrenergic stimuli, also play a role in the regulation of LES tone[26]. All these factors act in a complicated network.

Propofol might decrease the EGJ exposure by influencing both mechanisms. First, it is well established that propofol has a depressive effect on respiration[27, 28], which decreases diaphragmatic contractility and inhibits the pinchcock function of the diaphragm, further resulting in a negative effect on the EGJ exposure. Furthermore, although the regulation mechanism of propofol on LES is not yet clear, it was found that LES tone increased after the induction of anesthesia with propofol[29]. A previous study suggested that propofol could induce pronounced depression of the b-adrenergic related sympathetic nervous system[30]. The tone of LES is increased by beta-adrenergic antagonists, resulting in sphincter contraction[26].

Atropine is a classical anti-muscarinic widely used in clinical practice. In our study, we found that atropine can improve the extent of EGJ exposure during sedation EGD, as the only independent related factor in multivariate analysis (OR = 2.381, 95%CI: 1.297–4.371, *P* = 0.005). The explanation might be that the muscarinic agents enhance the LES tone and cause contraction through the parasympathetic nerve pathway[26]. This pathway is specifically blocked by atropine.

Regarding the different pathways, the negative effect of propofol on the extent of EGJ exposure was only partly antagonized. The exposure of EGJ was significantly better in the sedation group administered (2.64±1.05) than in the sedation group without atropine (score = 1.99±1.08), but still not as good as in the non-sedation group (score = 3.24±1.12,).

There are some limitations in our study. Regarding drug properties, the possible influence of fentanyl as a confounding factor has not been excluded. Due to the cross-sectional study design, it was neither randomized nor blinded. Due to ethical requirements, only one specific regular dose of atropine was administered in the study. More potential medicines of different mechanisms or different possible doses of atropine might be worth attempting in future work. Nearly all subjects were of the same race, and most of the study subjects were from Zhejiang Province, with similar genetic background and lifestyle, so that these results might not be generalizable to populations at the global level. The size of the study might also be a limitation. And further studies, probably multicenter and randomized are still needed.

In conclusion, our study suggested that the combination of propofol with fentanyl did reduce the extent exposure of the EGJ during EGD and reduced the detection of EGJ diseases. The application of atropine in the sedation endoscopic examination helped to improve the EGJ observation, but it could not achieve equal EGJ exposure for the non-sedation EGD.

## Supporting information

S1 Table Original information of subjects of the study. (XLS)

## References

[1] Jemal A, Siegel R, Xu J, Ward E. Cancer statistics, 2010. CA Cancer J Clin 2010;60:277–300 doi: 10.3322/caac.20073 20610543 10.3322/caac.20073

[2] Stein HJ, Feith M, Siewert JR. Cancer of the esophagogastric junction. Surg Oncol 2000;9:35–41 11525305 10.1016/s0960-7404(00)00021-9

[3] Zhou Y, Zhang Z, Zhang Z, Wu J, Ren D, Yan X, et al A rising trend of gastric cardia cancer in Gansu Province of China. Cancer Lett 2008;269:18–25 doi: 10.1016/j.canlet.2008.04.013 18501504 10.1016/j.canlet.2008.04.013

[4] Blaser MJ, Saito D. Trends in reported adenocarcinomas of the oesophagus and gastric cardia in Japan. Eur J Gastroenterol Hepatol 2002;14:107–113 11981333 10.1097/00042737-200202000-00003

[5] Ajani JA, D'Amico TA, Almhanna K, Bentrem DJ, Chao J, Das P, et al Gastric Cancer, Version 3.2016, NCCN Clinical Practice Guidelines in Oncology. J Natl Compr Canc Netw 2016;14:1286–1312 27697982 10.6004/jnccn.2016.0137

[6] Fitzgerald RC, di PM, Ragunath K, Ang Y, Kang JY, Watson P, et al British Society of Gastroenterology guidelines on the diagnosis and management of Barrett's oesophagus. Gut 2014;63:7–42 doi: 10.1136/gutjnl-2013-305372 24165758 10.1136/gutjnl-2013-305372

[7] Rantanen T, Oksala N, Sand J. Adenocarcinoma of the Oesophagus and Oesophagogastric Junction: Analysis of Incidence and Risk Factors. Anticancer Res 2016;36:2323–2329 27127139

[8] Bhardwaj A, McGarrity TJ, Stairs DB, Mani H. Barrett's Esophagus: Emerging Knowledge and Management Strategies. Patholog Res Int 2012;2012:814146 10.1155/2012/814146 PMC3369502 22701199

[9] Cohen LB, Hightower CD, Wood DA, Miller KM, Aisenberg J. Moderate level sedation during endoscopy: a prospective study using low-dose propofol, meperidine/fentanyl, and midazolam. Gastrointest Endosc 2004;59:795–803 15173791 10.1016/s0016-5107(04)00349-9

[10] Riphaus A, Rabofski M, Wehrmann T. Endoscopic sedation and monitoring practice in Germany: results from the first nationwide survey. Z Gastroenterol 2010;48:392–397 doi: 10.1055/s-0028-1109765 20140841 10.1055/s-0028-1109765

[11] Cohen LB, Wecsler JS, Gaetano JN, Benson AA, Miller KM, Durkalski V, et al Endoscopic sedation in the United States: results from a nationwide survey. Am J Gastroenterol 2006;101:967–974 doi: 10.1111/j.1572-0241.2006.00500.x 16573781 10.1111/j.1572-0241.2006.00500.x

[12] Wadhwa V, Issa D, Garg S, Lopez R, Sanaka MR, Vargo JJ. Similar Risk of Cardiopulmonary Adverse Events Between Propofol and Traditional Anesthesia for Gastrointestinal Endoscopy: A Systematic Review and Meta-analysis. Clin Gastroenterol Hepatol 2017;15(2):194–206 doi: 10.1016/j.cgh.2016.07.013 27451091 10.1016/j.cgh.2016.07.013

[13] Kim ES, Lee HY, Lee YJ, Min BR, Choi JH, Park KS, et al Negative impact of sedation on esophagogastric junction evaluation during esophagogastroduodenoscopy. World J Gastroenterol 2014;20:5527–5532 doi: 10.3748/wjg.v20.i18.5527 24833883 10.3748/wjg.v20.i18.5527 PMC4017068

[14] Japan Gastroenterological Endoscopy Society postgraduate education committee. Guidelines for Gastrointestinal Endoscopy. 3rd ed. Tokyo Igaku-Shoin Ltd.; 2006

[15] Zhaoshen L, Xiaoming Z, Shutian Z, Yiqi D, Jinbao L. Consensus of experts on sedation and anesthesia in the diagnosis and treatment of digestive endoscopy in China. Chin J Prac Intern Med. 2014;34:756–764

[16] Ramsay MA, Savege TM, Simpson BR, Goodwin R. Controlled sedation with alphaxalone-alphadolone. Br Med J 1974;2:656–659 4835444 10.1136/bmj.2.5920.656 PMC1613102

[17] Sharma P, Dent J, Armstrong D, Bergman JJ, Gossner L, Hoshihara Y, et al The development and validation of an endoscopic grading system for Barrett's esophagus: the Prague C & M criteria. Gastroenterology 2006;131:1392–1399 doi: 10.1053/j.gastro.2006.08.032 17101315 10.1053/j.gastro.2006.08.032

[18] Shaker R, Bardan E, Gu C, Massey BT, Sanders T, Kern MK, et al Effect of lower esophageal sphincter tone and crural diaphragm contraction on distensibility of the gastroesophageal junction in humans. Am J Physiol Gastrointest Liver Physiol 2004;287:G815–821 doi: 10.1152/ajpgi.00120.2004 15361362 10.1152/ajpgi.00120.2004

[19] Mittal RK, Shaffer HA, Parollisi S, Baggett L. Influence of breathing pattern on the esophagogastric junction pressure and esophageal transit. Am J Physiol 1995;269:G577–583 7485510 10.1152/ajpgi.1995.269.4.G577

[20] Mazur JM, Skinner DB, Jones EL, Zuidema GD. Effect of transabdominal vagotomy on the human gastroesophageal high-pressure zone. Surgery 1973;73:818–822 4703484

[21] Powley TL, Baronowsky EA, Gilbert JM, Hudson CN, Martin FN, Mason JK, et al Vagal afferent innervation of the lower esophageal sphincter. Auton Neurosci 2013;177:129–142 doi: 10.1016/j.autneu.2013.03.008 23583280 10.1016/j.autneu.2013.03.008 PMC3749274

[22] Farré R, Sifrim D. Regulation of basal tone, relaxation and contraction of the lower oesophageal sphincter. Relevance to drug discovery for oesophageal disorders. Br J Pharmacol 2008;153:858–869 doi: 10.1038/sj.bjp.0707572 17994108 10.1038/sj.bjp.0707572 PMC2267263

[23] Cotton BR, Smith G. The lower oesophageal sphincter and anaesthesia. Br J Anaesth 1984;56:37–46 6140931 10.1093/bja/56.1.37

[24] Sturdevant RA. Editorial: Is gastrin the major regulator of lower esophageal sphincter pressure. Gastroenterology 1974;67:551–553 4851520

[25] Cohen S, Lipshutz W. Hormonal regulation of human lower esophageal sphincter competence: interaction of gastrin and secretin. J Clin Invest 1971;50:449–454 doi: 10.1172/JCI106512 5540178 10.1172/JCI106512 PMC291941

[26] Preiksaitis HG, Tremblay L, Diamant NE. Cholinergic responses in the cat lower esophageal sphincter show regional variation. Gastroenterology 1994;106:381–388 8299905 10.1016/0016-5085(94)90596-7

[27] Physicians' Desk Reference 52nd ed. Problems and possible improvements. Montvale: Medical Economics Data Production Company 1998

[28] Muir WW, Gadawski JE. Respiratory depression and apnea induced by propofol in dogs. Am J Vet Res 1998;59:157–161 9492929

[29] Tournadre JP, Barclay M, Boulétreau P, Chassard D. Lower oesophageal sphincter tone increases after induction of anaesthesia in pigs with full stomach. Can J Anaesth 1998;45:479–482 doi: 10.1007/BF03012585 9598264 10.1007/BF03012585

[30] Horiguchi T, Nishikawa T. Heart rate response to intravenous atropine during propofol anesthesia. Anesth Analg 2002;95:389–392, table of contents 12145057 10.1097/00000539-200208000-00027

